# Evaluating the Accumulation of Antioxidant and Macro- and Trace Elements in *Vaccinium myrtillus* L.

**DOI:** 10.1007/s12011-021-02989-4

**Published:** 2021-10-29

**Authors:** Marta Kandziora-Ciupa, Marzena Dabioch, Aleksandra Nadgórska-Socha

**Affiliations:** 1 Ecology, Institute of Biology, Biotechnology and Environmental Protection, Faculty of Natural Sciences, University of Silesia in Katowice, Bankowa 9, 40-007 Katowice, Poland; 2 Department of Analytical Chemistry, Institute of Chemistry, Faculty of Science and Technology, University of Silesia in Katowice, Szkolna 7, 40-006 Katowice, Poland

**Keywords:** Bilberry, Antioxidants, Accumulation, Forest pollution, Toxic elements

## Abstract

This study was conducted in order to characterise the accumulation ability of *Vaccinium myrtillus* L for trace elements such as Al, Cd, Cu, Fe, Mn, Pb and Zn and selected macroelements Ca, K, Mg, Na and P. The accumulation of nutrient elements and trace elements (ANE and ATE) and changes in the ecophysiological responses in bilberry in differently polluted areas were compared. The accumulation of the elements in the roots, stems and leaves of bilberry from four sites (in the nearest vicinity of a zinc smelter, a Mining and Metallurgical Plant, a main road with a high traffic volume and an unprotected natural forest community) were measured using optical emission spectrometry with excitation using inductively coupled argon plasma after wet acid digestion. The highest Cd, Cu, Pb and Zn concentrations were found in the *V. myrtillus* samples that were growing under the influence of the emissions from the zinc smelter. Moreover, the level of the total accumulated trace metals (ATE—17.09 mmol_c_ kg^−1^) was also highest for the bilberry at this site. However, in the same area, the sum of the accumulated macronutrients (ANE—296.92 mmol_c_ kg^−1^) was lower than at the other sampling sites. An EF > 2 was found for Cd, Pb, Zn and Mn, which suggests that bilberries may be enriched in these metals. According to the translocation factor, *V. myrtillus* was an accumulator of Cd, Zn and Mn. An analysis of the ecophysiological responses showed that the greatest concentration of ascorbic acid was found in the leaves of *V. myrtillus* at the most contaminated site (3.32 mg g^−1^ fresh weight). There were no significant differences in the total phenols between the contaminated and non-contaminated sites. However, the lowest value of the total phenolic content (490.77 mg g^−1^ dry weight) was recorded at the site where the highest Fe concentration was detected in the leaves. A significantly positive correlation between the Cd, Pb and Zn concentrations and a strong negative correlation between the Mn concentration and ascorbic acid content in the leaves of bilberry was also observed. The results provide evidence that the ANE method, which is used to interpret the chemical composition of bilberry has made determining the impact of toxic trace metals on the mineral composition of *V. myrtillus* significantly easier and also that a non-enzymatic antioxidant such as ascorbic acid can be a good biomarker for determining the oxidative stress that is caused by toxic trace metals.

## Introduction

Forest ecosystems, especially those of central Europe and other regions, have been exposed to pollution for decades and are characterised by their high capacity to filter contaminants such as toxic trace metals that are transported within the atmosphere [1, 2]. The ground vegetation of forest ecosystems (defined as all terricolous plants—herbs, shrubs and trees) is included in the intensive monitoring of forest ecosystems in ICP Forests (International Co-operative Programme on Assessment and Monitoring of Air Pollution Effects on Forests), which is a particularly important element that provides information on any changes in a forest ecosystem and as a specific target for calculating critical loads/levels [3].

In the understorey of conifer forests in Europe and Northern Asia, *Vaccinium myrtillus* L. (bilberry) is a key and widely diffused long-lived clonal dwarf shrub species that belongs to the family Ericaceae and the genus *Vaccinium* [4–6]. It plays an essential role in nutrient cycling and is an important food source for many insects, birds and mammals, thus forming a link between the soil metal pool and the upper trophic levels in polluted environments [7]. Bilberry is a species that is relatively tolerant to a variety of environmental stressors such as long-term toxic trace metal pollution. At heavily polluted sites, *V. myrtillus* is able to grow, regenerate and spread [7–10], which enables bilberry to serve as model species of the forest floor of boreal forests that have been exposed to metal pollution. Additionally, bilberry can easily take up metals from the upper soil layer because its root system is generally shallow and this is the layer in which the metals from the atmosphere accumulate [7–9].

Anthropogenic trace elements are persistent and highly toxic pollutants that are serious risks to plants and animals and after they enter the food chain, also for humans [11, 12]. Toxic trace metals are listed as priority pollutants in many regions and ecosystems. They have been proven to be a great threat to plants; they affect the functions of many enzymes and proteins, disturb metabolism, exhibit phytotoxicity and eventually reduce growth due to physiological and biochemical processes [12, 13]. Elevated levels of toxic trace metals are associated with the increased generation of reactive oxygen species (ROS) [14] and oxidative stress, which leads, e.g., to the growth of membrane permeability and the leakage of ions, changes in the enzymatic activity, disturbances in the processes of photosynthesis and respiration, damage to cell protein, lipids and nucleic acid. In order to scavenge ROS and avoid oxidative damage, plants protect themselves with enzymatic and non-enzymatic (including ascorbic acid and phenolic compounds) mechanisms [15]. The responses of the antioxidant system/ecophysiological responses in plants can provide evidence of the early symptoms of the damage, which precede the morphological or ultrastructural damage that is caused by the metals. Therefore, early environmental stress could be estimated based on ecophysiological responses of a plant, which might be useful in toxic trace element pollution biomonitoring [16, 17]. Understanding the mechanisms that underlie the resistance or tolerance of plants to different stress factors, including toxic trace metals, is extremely important in the era of global warming, where the mobility of pollutants in the environment increases [18].

It is, therefore, reasonable to find and select the most useful indicators that are applicable in the bioindication of a forest environment. The research hypothesis of this study assumes that an environmental contamination with trace elements influences the pattern of accumulated elements and causes changes in a plant’s antioxidants, which can be used in the bioindication of forest ecosystems. Therefore, this study was conducted to characterise the accumulation ability of *Vaccinium myrtillus* L. We compared the accumulation of nutrient elements and trace elements (ANE and ATE) and changes in the ecophysiological responses in bilberry in differently polluted areas.

The detailed objectives of this study were (1) to document and compare the nutrient (ANE) and trace element (ATE) concentrations in the leaves, stems and roots of *V. myrtillus* that was growing on metal-polluted and relatively unpolluted sites, (2) to evaluate and compare the distribution patterns and the accumulation efficiency of toxic trace metals in *V. myrtillus* and (3) to estimate the ecophysiological responses to stress that are caused by toxic trace metals in the leaves of bilberry growing under field conditions.

## Material and Methods

### Study Area

**Fig. 1:**
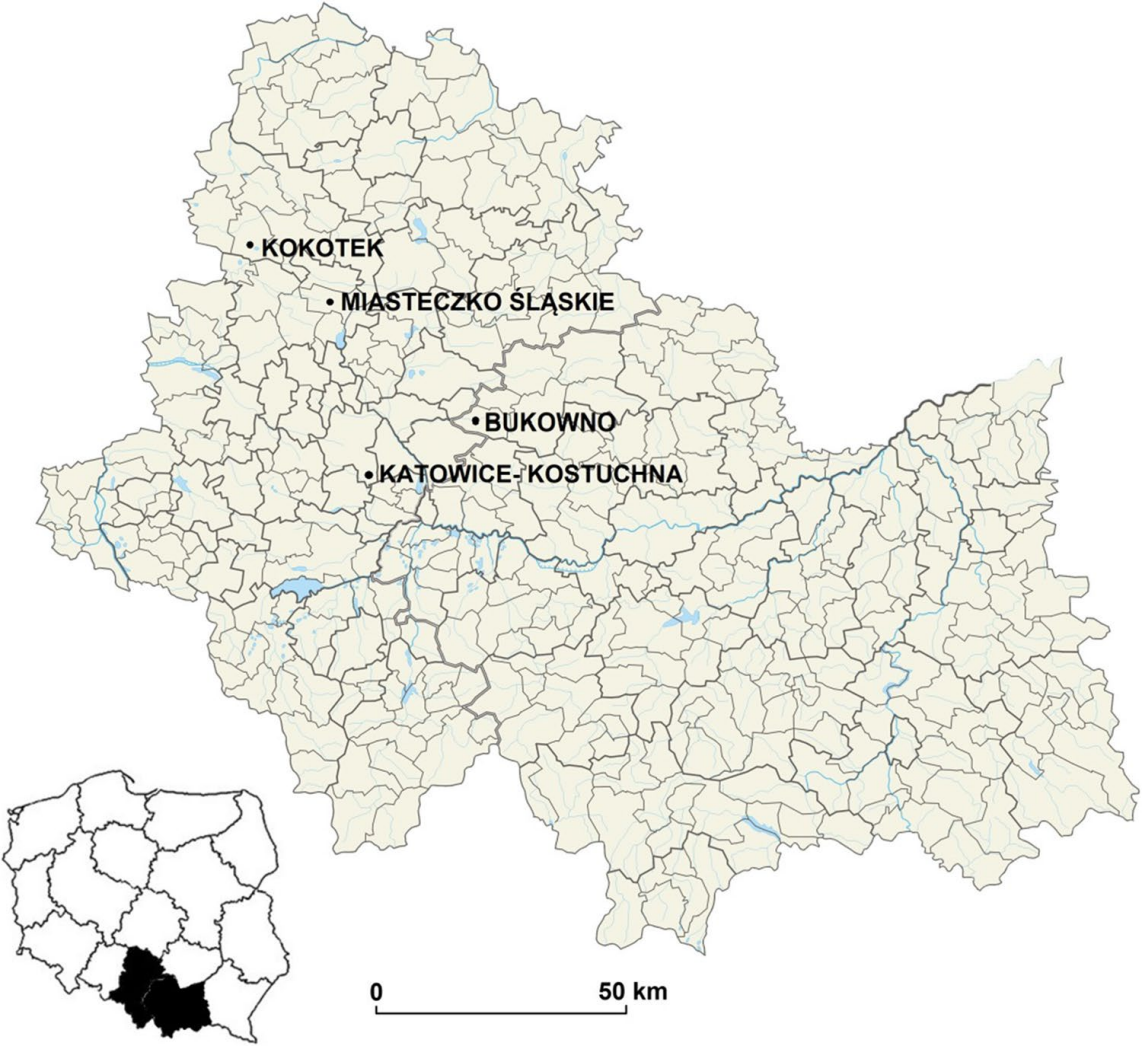
Location map of sampling sites (Kandziora-Ciupa et al. 2021)

The study was performed around a middle-aged Scots pine (*Pinus sylvestris* L.) forest, which has mixed stands with birch (*Betula pendula* L.), European beech (*Fagus sylvatica* L.) and pedunculate oak (*Quercus robur* L.) and sandy acidic soils. The plant materials was collected from three differently polluted sites (in the nearest vicinity of the zinc smelter “Miasteczko Śląskie” in Miasteczko Śląskie (active since 1968) at 50°31′22.655′′ N 18°56′8.699′′ E (M), in the nearest vicinity of the ZGH “Boleslaw” Mining and Metallurgical Plant in Bukowno (active since 1955) at 50°15′55.6′′ N 19°26′34.64′′ E (B), in Katowice-Kostuchna in the vicinity of a main road with a high traffic volume at 50°11′42.75′′ N 19°0′26.363′′ E (K) and in an unprotected natural forest community in Kokotek at 50°36′21.287′′ N 18°42′59.806′′ E (KO), which was considered the control site. All of the sites are in the Śląskie or Małopolskie provinces in southern Poland (Fig. 1) [19].

### Sample Collection

*Vaccinium myrtillus* L. leaves, stems and roots were collected in May 2017. Each sampling site consisted of a 25 × 25 m square from within which three replicates of fully mature and undamaged leaves, stems and roots were collected from at least 20 different bilberry specimens. After collection, the samples were placed in plastic bags, deposited on ice and transported to the laboratory.

### Analysis of Macro- and Toxic Trace Element Concentration in the Plant Samples

In order to determine the macroelement (Ca, K, Mg, Na, P) and toxic trace element (Al, Cd, Cu, Pb, Zn, Fe, Mn) concentrations in the samples of bilberry (leaves, stems and roots), the plant material was washed with tap water to remove any substrate and dust deposits, then rinsed twice with distilled water and dried at 105 °C. A 0.25-g portion of dry plant material in digestion vessels was treated with 4 ml concentrated nitric acid and was then pre-digested for 24 h at room temperature. Next, the tubes were placed in digital dry baths (The Labnet Digital Dry Baths, dual block; temperature range: + 5º above ambient temperature to 150º C; temperature uniformity: ± 0.2 °C) and heated to 120 °C until the samples were completely digested. After cooling to room temperature, the digested samples were filtered into 50 ml plastic bottles and diluted to 25 ml with deionised water [20]. The levels of the toxic trace elements and macroelements were measured using optical emission spectrometry with excitation using inductively coupled argon plasma (SPECTROBLUE ICP-OES, Spectro Analytical Instruments, Germany). The optimum measurement conditions are listed in Table 1. The wavelength, LOD (limit of detection), LOQ (limit of quantification) and *R*^2^ (correlation coefficients) for the determined elements using the ICP-OES method in this study are listed in Table 2. The quality of the analytical procedure was determined using a reference material (Certified reference Material CTA-OTL-1 Oriental Tobacco Leaves) with the same quantities of samples.

**Table 1:** Measurement conditions for ICP-OES

Rf power, kW	1.4
Frequency, MHz	27.12
Plasma torch	Quartz
Plasma gas flow, L min^−1^	12.0
Auxiliary gas flow, L min^−1^	0.8
Nebulizer gas flow, L min^−1^	0.8
Crossflow nebulizer, bar	2.4
Sample uptake mL min^−1^	1
Number of replicates	3
Read delay time, s	3
Scope of the polichromator, nm	165–285 285–470
Holographic grid, grooves mm^−1^	3600 1800
Integration time, s	3

**Table 2:** The wavelength, LOD, LOQ, and *R*^2^ for the determined elements

Element	Wavelength (nm)	LOD	LOQ	*R*^2^
Al	176.641	0.0092	0.0183	0.99997
Ca	422.673	0.0090	0.0181	0.99996
Cd	214.438	0.0003	0.0007	0.99995
Cu	324.754	0.0053	0.0106	0.99990
Fe	275.573	0.0091	0.0182	0.99997
K	766.491	0.1007	0.2015	0.99999
Mg	279.553	0.0005	0.0011	0.99982
Mn	259.373	0.0008	0.0016	0.99998
Na	589.592	0.0009	0.0018	0.99992
P	178.287	0.0316	0.0632	0.99994
Pb	168.215	0.0072	0.0143	0.99989
Zn	213.856	0.0092	0.0184	0.99996

*LOD* limit of detection, *LOQ* limit of quantification, *R*^*2*^ correlation coefficients

### The Accumulation of the Nutrient Elements and the Trace Elements

The accumulation of the nutrient elements and the trace elements (ANE and ATE) in the studied plants was analysed according to the method by [21]. To calculate the sum of the elements, the values describing the amount of each element were converted into equivalents. The sum of the elements in mmol_c_ kg^−1^ was calculated according to the following formula:

$$Y=\sum_{i=1}^{i}\frac{Z}{z}$$

where Z is the element content in mg kg^−1^ and *z* is the atomic mass/valence of an ion

$$Y=\frac{N}{14}+\frac{P}{31}+\frac{K}{39}+\dots +\frac{Cu}{31.8}+\frac{Mn}{27.5}+\dots$$

After estimating the sum of the accumulation, the percentage of each element (*X*) was calculated:

$${X}_{z}=\frac{\left(\frac{Z}{z}\right)\times 100}{Y}$$

### Metal Accumulation Efficiency

To evaluate the metal accumulation in the plant samples, the translocation factor (TF) and enrichment factor (EF) were calculated, results were expressed on dried weight. The TF is the ratio of metal concentration in the shoots (leaves + stems) and the roots. A TF > 1 indicates that a plant translocates metals effectively from the roots to the shoot [22]. The EF shows the degree of the accumulation of toxic trace metals in the plants growing at the contaminated sites compared to those growing at the non-contaminated sites. The EF was calculated as EF = C/C where C and C refer to the metal concentrations (mg kg^−1^) in the *Vaccinium myrtillus* shoots (leaves + stems) from the contaminated and the non-contaminated site [22]. The values of the factors mentioned above help to specify the strategy of metal accumulation in the plants and were calculated for the main metals (Cd, Mn, Pb and Zn),

### Analysis of the Ecophysiological Parameters of the Plants

The estimation of the reducing capacity of the plants, as expressed by the total phenolic content, might indicate the antioxidative potential and the resistance of the plants to environmental stress. The soluble phenolics were determined by homogenizing a fresh leaf (0.3 g) at 4 °C in 80% methanol to a final volume of 3 ml and then centrifuged at 12,000 × *g* for 15 min at 4 °C. The assay mixture contained 30 μl of the supernatant, 470 μl of redistilled water, 975 μl of 2% Na_2_CO_3_ and 25 μl of a 2-N Folin-Ciocalteu reagent. The samples were incubated for 1 h at 45 °C and the absorbance was measured at 750 nm. The total phenol content was calculated based on a standard curve that was prepared using gallic acid [23].

The ascorbic acid content was calculated using the formula given by [24]:

$$\mathrm{Ascorbic acid }\left(\mathrm{mg}\times {\mathrm{g}}^{-1}\mathrm{ fresh weight}\right)=\frac{\left({\mathrm{E}}_{\mathrm{o}}-{\mathrm{E}}_{\mathrm{s}}-{\mathrm{E}}_{\mathrm{t}}\right)\mathrm{V}}{\mathrm{W}\times 100}\times 100$$

where *V* is the volume of the extract, *W* is the weight of the fresh leaf sample (g) and *E*_o_, *E*_s_ and *E*_t_ are the optical densities of a blank sample, a plant sample, and a sample with ascorbic acid, respectively.

### Statistical Analysis

The data concerning macroelements, toxic trace elements and other ecophysiological parameters were examined for normality and homogeneity of variance. When there was a normal distribution and variance homogeneity, the data was analysed using ANOVA and the treatments were treated as the independent variables. Any significant statistical differences of all of the variables were established using the Tukey`s test (ANOVA; Statistica 10 package). Pearson`s correlation coefficient was calculated to assess the relationship between the estimated metal concentrations and the ecophysiological parameters in the bilberry leaves. CANOCO 4.5 was used to perform the principal component analysis, which assessed the similarities and relationships between the metal concentrations and the ecophysiological parameters in the studied areas.

## Results

The results of the bioaccumulation of the trace metals and macroelements that were analysed in the leaves, stems and roots of *V. myrtillus* are listed in Tables 3 and 4. Generally, the levels of the toxic trace element were significantly higher in the most polluted area (M). An exception to this was Mn, which had markedly higher values at the Katowice-Kostuchna site (K). The levels of the toxic trace elements (Al, Cd, Cu, Fe, Mn, Pb, Zn) in the different *V. myrtillus* organs from the most polluted area followed the general pattern: roots > stems > leaves (Table 4). Again, Mn was an exception because at the site where its highest concentration was found (K), this pattern was completely the opposite: leaves > stems > roots (Table 4).

**Table 3:** The concentrations of macroelement (mg kg^**−**1^ d.w.) in the various plant tissue of *V. myrtillus* (mean values)

	Ca	K	Mg	Na	P
Leaves					
M	2938.77 a	3460.94 b	520.47 a	1.89 a	3276.70 a
B	3789.63 ab	3900.60 c	1057.99 b	2.21 a	3128.49 a
K	3638.58 ab	4991.42 d	1220.66 c	1.70 a	3174.06 a
KO	4008.55 b	3309.94 a	1331.88 d	1.58 a	3324.81 a
Stems					
M	4402.27 b	1337.38 a	435.89 a	2.51 b	1400.63 a
B	6466.70 c	1672.39 b	936.66 b	2.99 b	1581.34 a
K	2124.61 a	2487.89 c	526.77 a	2.76 b	1866.24 a
KO	4794.03 bc	1881.48 b	838.31 b	1.88 a	2244.97 b
Roots					
M	1749.80 b	817.42 b	228.95 a	1.54 a	1038.79 b
B	1062.78 a	950.02 a	240.07 ab	2.11 b	830.16 a
K	1075.66 a	1452.52 c	287.27 b	2.90 c	1059.97 b
KO	1383.15 ab	959.96 a	263.11 ab	1.72 a	995.62 b

The different letters denote significant differences between the macroelement concentrations in the same organ (*p* < 0.05)

*M* Miasteczko Śląskie. *B* Bukowno, *K* Katowice-Kostuchna, *KO* Kokotek

**Table 4:** The concentrations of trace elements (mg kg^**−**1^ d.w.) in the various plant tissue of *V. myrtillus* (mean values)

	Al	Cd	Cu	Fe	Mn	Pb	Zn
Leaves							
M	107.17 a	9.81 b	9.06 b	108.58 b	39.91 a	232.08 b	332.26 b
B	148.47 b	0.34 a	5.78 ab	310.97d	282.74 b	17.86 a	120.24 a
K	175.94 c	0.41 a	2.17 ab	141.42c	1124.81 d	4.62 a	12.17 a
KO	98.23 a	0.46 a	3.62 a	59.57a	428.07 c	4.55 a	16.36 a
Stems							
M	181.82 a	17.16 b	11.13 c	133.39 b	66.85 a	390.44 b	737.85 c
B	140.30 a	0.84 a	2.33 b	238.71 c	380.32 b	22.20 a	361.66 b
K	142.13 a	0.53 a	0.69 a	95.57 a	760.73 d	6.50 a	25.20 a
KO	119.71 a	0.50 a	2.05 ab	56.40 a	507.17 c	6.91 a	72.27 a
Roots							
M	214.38 c	26.51 b	18.82 b	264.66 b	93.30 a	668.31 b	1003.10 b
B	129.33 b	0.29 a	1.59 a	117.87 a	160.33 b	32.43 a	126.40 a
K	196.48 b	0.37 a	1.14 a	151.27 a	429.46 d	28.62 a	15.33 a
KO	113.19 a	1.29 a	2.29 a	101.08 a	204.56 c	48.27 a	66.23 a

The different letters denote significant differences between the element concentrations in the same organ (*p* < 0.05)

*M* Miasteczko Śląskie, *B* Bukowno, *K* Katowice-Kostuchna, *KO* Kokotek

The accumulation of the nutrient and trace elements (ANE and ATE) in the *V. myrtillus* shoots was calculated separately for the macro- and toxic trace elements. At the most contaminated site (M), the average level of accumulated macronutrients was lowest compared to the other areas (Table 5). A higher, almost two-fold, value of ANE was found for the *V. myrtillus* at B, K and KO sites (Table 5). Quite the opposite was found in the case of the total accumulated trace metals (ATE), where a higher value was detected for the *V. myrtillus* at the contaminated sites, especially at Miasteczko Śląskie (three-fold higher than at the non-contaminated site in Kokotek-KO) (Table 6). At the most contaminated site (M), the percentages of Cd, Pb and Zn were higher than the percentages that were obtained for the bilberry that had been collected at the other sites (Table 6).

**Table 5:** Accumulation of nutrient elements (ANE) in *V. myrtillus* shoots

Stands	ANE macroelements [mmol_c_ kg^**−**1^]	Ca [%]	K [%]	Mg [%]	Na [%]	P [%]
M	296.92	13.74	16.59	4.03	0.02	19.83
B	463.98	14.04	14.81	6.49	0.01	15.00
K	462.21	14.61	20.55	8.12	0.01	16.50
KO	467.40	15.37	13.01	8.45	0.01	16.50

*M* Miasteczko Śląskie, *B* Bukowno, *K* Katowice-Kostuchna, *KO* Kokotek

**Table 6:** Accumulation of trace elements (ATE) in *V. myrtillus* shoots

Stands	ATE trace elements [mmol_c_ kg^**−**1^]	Al [%]	Cd [%]	Cu [%]	Fe [%]	Mn [%]	Pb [%]	Zn [%]
M	17.09	0.74	0.016	0.0003	0.36	0.14	0.21	0.95
B	11.97	0.82	0.0004	0.0001	0.83	0.76	0.01	0.27
K	8.53	1.05	0.0006	0.0001	0.41	3.30	0.004	0.03
KO	5.11	0.56	0.0006	0.0001	0.16	1.20	0.003	0.04

*M* Miasteczko Śląskie, *B* Bukowno, *K* Katowice-Kostuchna, *KO* Kokotek

The mean values of the translocation factor (TF) and enrichment factor (EF) for bilberry are listed in Table 7. A translocation factor above 1 (TF > 1) in *V. myrtillus* was observed for almost all of the investigated metals at all of the areas; the exceptions were Pb, for which TF > 1 was found for only one sampling site (B, Table 7) and Cd, for which TF was below one at only the non-contaminated site (KO) (Table 7). The highest EF values for Cd (28.1), Pb (54.3) and Zn (12.07) were detected for the *V. myrtillus* at site M and the highest EF for Mn (2.02) was found for the bilberry at Katowice-Kostuchna (site K; Table 7).

**Table 7:** Translocation factor (TF) and Enrichment factor (EF) in *V*. *myrtillus* shoots

Stands	Cd	Mn	Pb	Zn
Translocation factor				
M	1.02	1.14	0.93	1.07
B	4.04	4.14	1.24	3.81
K	2.53	4.39	0.39	2.44
KO	0.74	4.57	0.24	1.34
Enrichment factor				
M	28.01	0.11	54.30	12.07
B	1.23	0.71	3.49	5.44
K	0.97	2.02	0.97	0.42
KO	1.00	1.00	1.00	1.00

*M* Miasteczko Śląskie, *B* Bukowno, *K* Katowice-Kostuchna, *KO* Kokotek

**Fig. 2:**
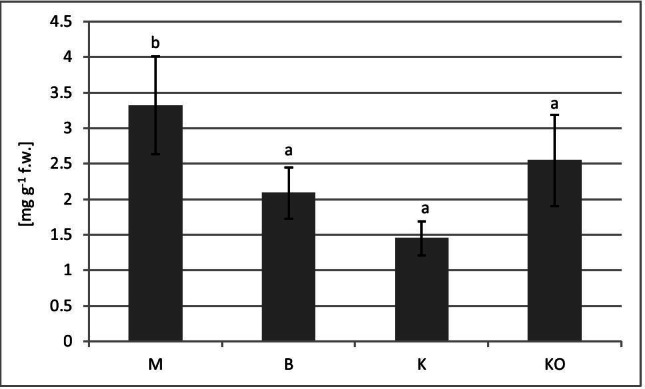
Ascorbic acid contents (mg g^−1^ fresh weight) in *V. myrtillus* leaves (mean values ± SD). Different letters above the columns indicate significant differences (*p* < 0.05). M–Miasteczko Śląskie, B–Bukowno, K–Katowice-Kostuchna, KO–Kokotek

The greatest concentration of ascorbic acid was detected in the leaves of the *V. myrtillus* at the most contaminated M site (3.32 mg g^−1^ fresh weight) (Fig. 2). This dependence was confirmed by a positive correlation between the ascorbic acid content and the concentrations of Cd, Pb and Zn in the bilberry leaves (Table 8 and Fig. 4). The lowest content of ascorbic acid was detected at site K (Fig. 2). There was also a strong negative correlation between both the Mn and Mg and AA content in the bilberry leaves (Table 8 and Fig. 4).

**Table 8:** The correlation coefficients between metal concentration and ecophysiological parameters in the *V. myrtillus* leaves (*p* < 0.05)

	Al	Ca	Cd	Cu	Fe	K	Mg	Mn	Na	P	Pb	Zn
Ascorbic acid	NS	NS	0.92^*^	NS	NS	NS	− 0.80*	− 0.70*	NS	NS	0.92*	0.92*
Soluble phenolics	NS	NS	NS	NS	− 0.82*	NS	NS	NS	NS	NS	NS	NS

*NS* not significant

^*^Significant

**Fig. 3:**
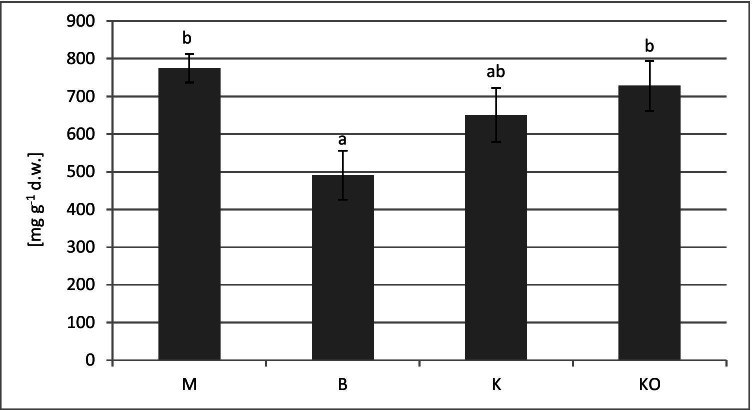
Changes of soluble phenolics (mg g^−1^ dry weight) in *V. myrtillus* leaves (mean values ± SD). Different letters above the columns indicate significant differences (*p* < 0.05). M–Miasteczko Śląskie, B–Bukowno, K–Katowice-Kostuchna, KO–Kokotek

Although there were no significant differences in the total phenols between the contaminated and non-contaminated sites (Fig. 3), the lowest value of the total phenolic content (490.77 mg g^−1^ d.w.) was recorded at Bukowno (B), where the highest Fe concentration was detected in the leaves (Table 4 and Fig. 3). Finally, there were significant negative correlations between the total phenolic content and the concentrations of the Fe were found in the bilberry leaves (Table 8).

**Fig. 4:**
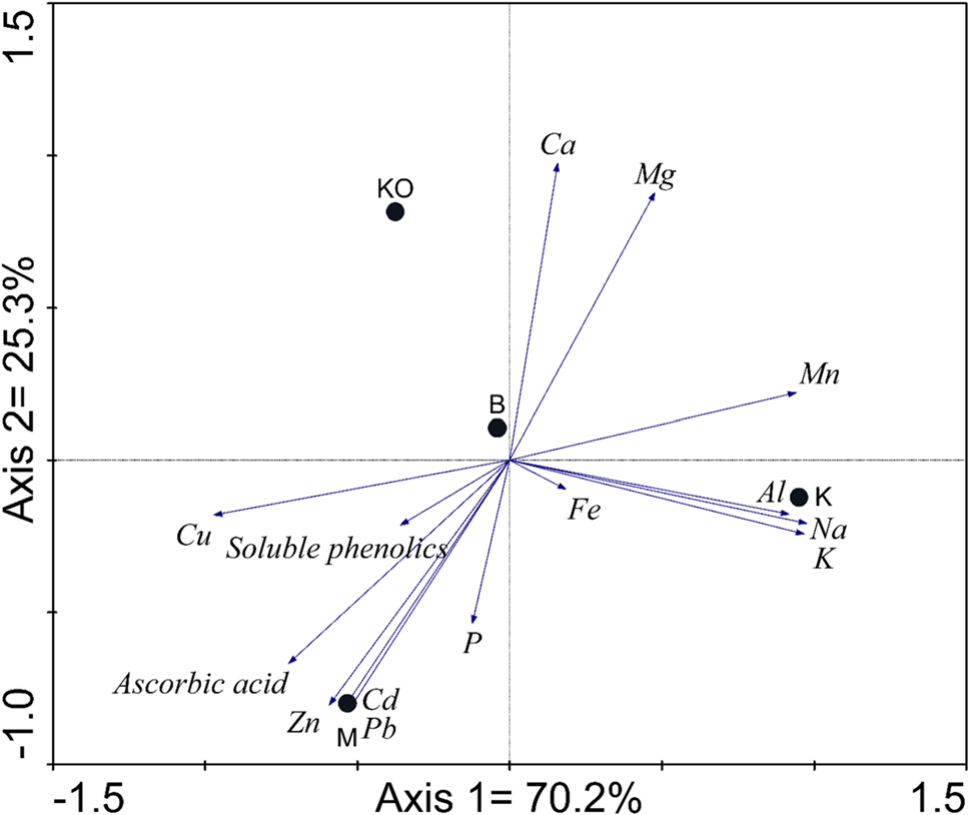
Principal component analysis (PCA) biplot of sampling sites and heavy metal concentrations and ecophysiological parameters*.* M–Miasteczko Śląskie, B–Bukowno, K–Katowice-Kostuchna, KO–Kokotek

In the PCA for the antioxidant analysis of this study, there were two axes of PCA that explained 95.5% (70.2% by axis 1; 25.3% by axis 2) of the variability for the ecophysiological responses of the *Vaccinium myrtillus* leaves (Fig. 4).

## Discussion

The results of study showed that Vaccinium myrtillus appears to be a good bioindicator for metal pollution because it has higher values in the areas that are polluted with the most toxic trace elements with the exception of Mn (see also [7, 25]. The highest concentrations of toxic trace elements were usually much higher than the values that are considered to be sufficient or normal and they exceeded the toxic levels for plants (according to [26, 27]. Other field studies in heavily polluted areas near a Ni-Cu smelter showed that bilberry was tolerant to high metal levels and was also not significantly stressed [28]. In this study, the TF > 1 for Mn (highest values) and Zn was observed at all investigated sites except for Cd at the control site (Kokotek-KO). A TF factor > 1 indicates that a plant efficiently translocates metals from the roots to the shoots. According to [29, 30] and [31], a TF > 1 indicates a metal extraction potential, which, according to [32], makes such plants effective phytoremediators. TF values higher than 1 mean that a plant acts as an accumulator [31], which is particularly evident in the case of Mn. Many authors (e.g., [6, 7, 9, 25, 33] have confirmed the ability of *V. myrtillus* to readily accumulate Mn. The relatively low TF values of bilberry for Pb may result from that it is a toxic trace metal with limited mobility that is first accumulated in the roots. Additionally, low TF values for this metal indicate its potential to stabilise in the soil [34].

The highest enrichment factors in bilberry were found for Pb. Additionally, an EF > 2 was observed for Zn, Cd and Mn, which, according to [35], means that bilberry might be enriched in these metals; this enrichment is mainly associated with their presence for many years in the vicinity of the activity of the zinc smelter and Mining and Metallurgical Plant and the high concentrations of Cd, Pb and Zn in the upper soil layers in these areas.

The relationship between the environment and bilberry that is based on the content of the individual components in a plant should be treated as approximate. Therefore, it is necessary to acquire new methods for interpreting the chemical composition of plants, which will permit a more precise description of the relationship between a plant and the environment. Such methods include the ANE (accumulation of nutrient elements) thanks to which it is possible to characterise the habitat richness with the components that are available to plants and the state of supplying plants with minerals, which would be much better than using the conventional interpretation of the content of each component separately [21, 32]. In this study, unambiguously interpreting the content of macro-elements in *V. myrtillus* was difficult. Only the ANE method permitted the observation that in the same area in which the highest value of the sum of the trace elements (and percentage of Cd, Pb, Zn) was found, the sum of the accumulated macronutrients was definitely lower compared to the other sampling sites. The content of macro-elements in the bilberry shoots, which was interpreted using the ANE method, indicated that site M (near the zinc smelter) is a habitat that is less rich in nutrients than the other areas that were selected for the research.

In plants that are exposed to toxic trace metals, various non-enzymatic antioxidants accumulate and these are involved in detoxifying the trace metal and ROS-scavenging pathways [36]. Antioxidant molecules including ascorbic acid and phenolic compounds inhibit oxidation and play crucial roles in the stress responses [37, 38].

Apart from the basic role of ascorbic acid (AsA) in protecting plants from the deleterious effects of ROS, AsA also plays an essential role in the growth and normal functioning of plants, it protects lipids and proteins and induces plant growth, photosynthesis, transpiration and photosynthetic pigments [39–41]. Increased levels of Cd, Pb and Zn generally resulted in a higher ascorbic acid content in the leaves of *V. myrtillus*, and these results are consistent with previous studies on the accumulation of toxic trace metals in *V. vitis-idaea* [6]. A similar relationship between the increase in AsA content due to the increased concentration of various toxic trace metals was also found by [42–44]; these results indicated that increased levels of ascorbic acid participate in the plant responses to toxic trace metal accumulation and have a positive effect on pollution tolerance by plants [45, 46]. Simultaneously, an excess Mn concentration significantly reduced the content of ascorbic acid. This phenomenon has been observed by many authors [6, 42, 46–49], which suggests that AsA might be one of the most important compounds in the response to Mn stress and the adaptation of plants to the environment, especially for bilberry, which has been reported to accumulate high levels of Mn. The decrease in the ascorbic acid content in response to Mn stress could be explained by the fact that ascorbic acid and its metabolic precursors give rise to oxalic acid; oxalate is involved in the detoxification of toxic metals [42, 50, 51] and could enhance the defences against Mn stress in bilberry leaves.

The leaves of *V. myrtillus* are a rich source of a variety of secondary metabolites such as phenolic compounds, which are responsible for the adaptation of plants to the environment [16]. Plants can synthesise phenolic compounds as a protective mechanism against adverse environmental conditions in order to remove ROS from reduce oxidative stress and chelate toxic metals through functional groups, both hydroxyl and carboxylic acid [52, 53]. Additionally, phenolic compounds can participate in the ROS-scavenging mechanisms by cooperating with the antioxidant enzymes, especially with the peroxidases and superoxide dismutase [23, 52]. Many authors [16, 38, 52, 53] have reported that exposure to toxic trace metals increases the production of phenolic compounds in plants. However, in this study, the level of phenolic compounds did not increase in the presence of the investigated toxic trace metals. Additionally, a decrease of phenolic compounds under Fe contamination was observed. Moreover, [54] found a decrease in the phenol content in Cd-treated okra plants. As [55] suggested, the lack of an increase of phenolic compounds in blueberry leaves might have been caused by an excessive accumulation of toxic metals, especially Cd, Pb and Zn, which could impair the antioxidative system responses based on phenolics in such way that plants are not able to synthesise new phenols. Additionally, [55] reported that the highest cadmium exposure did not significantly affect the total phenolic compounds in *Erica andevalensis* leaves and [56] they did not observe an increase of the total phenolic compounds in *Spartina densiflora* under high cadmium exposure. However, as in this study, they found an increased ascorbic acid content in response to cadmium.

## Conclusions

The results confirmed that *Vaccinium myrtillus* L. is one of the species of the conifer forest understorey that can grow in metal-polluted areas and that the contamination changes its ecophysiological responses. An EF > 2 was found for Cd, Pb, Zn and Mn, which suggests that bilberries might be enriched in these metals and could act as indicators of metal contamination of the soil. According to the translocation factor, *V. myrtillus* acted as an accumulator of Cd, Zn and especially for Mn, which makes this species good for phytoremediation.

The increased content of toxic trace metals caused disturbances in the mutual relationships of the examined elements and had a negative effect on the mineral composition of *V. myrtillus*.

This study suggests that a non-enzymatic antioxidant such as ascorbic acid could be a good biomarker for the oxidative stress that is caused by toxic metals and could be used as a warning indicator for forest ecosystems. However, the role of various types of antioxidants in detoxifying toxic trace metals has not yet been fully explained, especially for plants that are growing in natural conditions in which metals do not act as a single factor. Therefore, it is important to conduct this type of investigation to identify vital clues for improving pollution biomonitoring in forest ecosystems.

## Data Availability

The data presented in this study are available on request from the corresponding author. The data are not publicly available due to privacy restrictions.
